# Pregnancy and Bariatric Surgery: Significant Variation in Bariatric Surgeons' Practices and Preferences: A National Survey

**DOI:** 10.1089/bari.2021.0045

**Published:** 2022-06-08

**Authors:** Daniëlle S. Bonouvrie, Sophie B.M. Taverne, Loes Janssen, Arijan A.P.M. Luijten, François M.H. van Dielen, Wouter K.G. Leclercq

**Affiliations:** ^1^ Obesity Center Máxima, Máxima Medical Center, Eindhoven/Veldhoven, The Netherlands.; ^2^ NUTRIM School of Nutrition and Translational Research in Metabolism, Maastricht University, Maastricht, The Netherlands.; ^3^ Department of Surgery, Maastricht University Medical Center, Maastricht, The Netherlands.; ^4^ Department of Surgery, Maxima Medical Center, Eindhoven/Veldhoven, The Netherlands.

**Keywords:** bariatric surgery, pregnancy, fertile women, counseling, education, preferences

## Abstract

**Background:**

Bariatric complications may occur during pregnancy, potentially causing serious maternal and fetal problems. The aim of this study was to determine the current practice and preferences of bariatric surgeons regarding the pregnancy care of fertile women before and after bariatric surgery.

**Methods:**

A 26-question anonymous online survey was designed and sent to all bariatric surgeons of the Dutch Society of Metabolic and Bariatric Surgery.

**Results:**

At least one bariatric surgeon from each bariatric center (*n* = 18) completed the survey. In case of a future child, wish sleeve gastrectomy became more popular than Roux-en-Y gastric bypass. All surgeons provided preoperative education regarding bariatric complications during pregnancy. Nine centers without neonatal intensive care would not refer pregnant women with acute complications. Half of the centers had a standard operating procedure. Seven per 18 bariatric centers had seen at least one postbariatric pregnant patient with severe maternal morbidity. One case of perinatal mortality was reported.

**Conclusion:**

There is an inconsistent and often below guideline standard daily practice regarding pregnancy before and after bariatric surgery. There is limited experience with pregnant women with acute bariatric complications. Referral to tertiary centers is inadequate. Better information provision for both professionals and patients regarding possible complications is needed.

## Introduction

Morbid obesity is known to negatively affect fertility, to increase the risk of complications during pregnancy and childbirth, and to enhance the chance of adverse perinatal outcomes.^1–4^ Bariatric surgery (BS) is the most effective long-term treatment for morbid obesity.^5^ It also results in improved pregnancy-related outcomes,^6–8^ which contribute to the increase in bariatric procedures performed in fertile women.

However, BS is also associated with various maternal and fetal risks. Over the last few years, more studies have become available regarding acute small bowel obstruction during pregnancy due to internal herniation or intussusception, especially after Roux-en-Y gastric bypass (RYGB).^9–14^ The diagnosis of these acute abdominal bariatric complications during pregnancy can be challenging because the clinical presentation might be similar to general pregnancy-related complaints and imaging techniques lack high sensitivity, specificity, or availability.^13^ Furthermore, the incidence is low, whereas expertise is needed for adequate clinical decision-making.^11–13^

Related to the increase in evidence of pregnancy-related risks after BS, various guideline articles with recommendations regarding the care of pregnant women after BS have recently been published.^15,16^ Recommendations are, among others, to postpone a pregnancy after BS for 12–24 months so that maternal weight has been stabilized, to prescribe specific supplementation during the preconception and periconception period, and to avoid excessive gestational weight gain.^9,15,16^ Recommendations regarding the diagnosis and treatment of acute abdominal bariatric complications are limited.^15,16^ Moreover, it is unclear to what extent these guideline articles and recommendations are implemented in daily practice.

In 2018 and 2019, respectively, 29.7% and 30.0% of the patients who underwent BS in our clinic were women of childbearing age (18–40 years). As a tertiary referral center, we have implemented various changes to improve the care for mother and child (Appendix A1).

The aim of this study was not only to get insight in the preoperative education regarding pregnancy-related outcomes of fertile women undergoing BS but also in the current practice and preferences toward the care and referral of (non)pregnant women with a history of BS and possible acute abdominal complications in the Dutch bariatric care.

## Materials and Methods

### Ethics and informed consent

The local institutional review board waived the offical review and the documentation of informed consent as this study was regarded a descriptive questionaire study.

### Study population

All bariatric surgeons of the Dutch Society of Metabolic and Bariatric Surgery were invited to participate in the survey. There are 18 bariatric centers in the Netherlands with only one bariatric center located in a facility with an obstetric high care (OHC) and a neonatal intensive care unit (NICU). There are no academic centers with a bariatric center.

### Survey

An anonymous survey was designed using an online platform for questionnaires and surveys (Survey Monkey Inc., San Mateo, CA). The survey consisted of 26 questions regarding the care of pregnant women or women with an active child wish, just before or after BS (Appendix A2). Seventeen questions addressed individual surgeon's practice and preferences, whereas nine questions were focused on visualizing current practice within the bariatric center.

The 26 items consisted of 10 dichotomous, 2 open, and 14 multiple-choice questions. One open question required at least three answers; all other questions required only one. Some questions allowed textual remarks. In two questions, conditional branching was used, creating a custom path for the respondents through the survey. The number of questions therefore varied between 25 and 26 questions.

The online survey was collected between April and June 2019. Reminders to participate in the survey were sent 4 and 9 weeks after the initial invitation.

### Analysis

All completed surveys were included and analyzed. Questions that addressed individual surgeon's practice and preferences were analyzed at surgeon level. Questions that addressed current practice within a bariatric center were analyzed at center level, that is, one answer was included for each center. For these latter analyses, discrepant answers by surgeons working at the same bariatric center were discarded. Categorical variables are presented as number only or as number (percentage). Continuous data are presented as mean (range, minimum–maximum). Descriptive statistics were calculated with IBM SPSS statistic software, version 24.0.

## Results

**FIG. 1.**
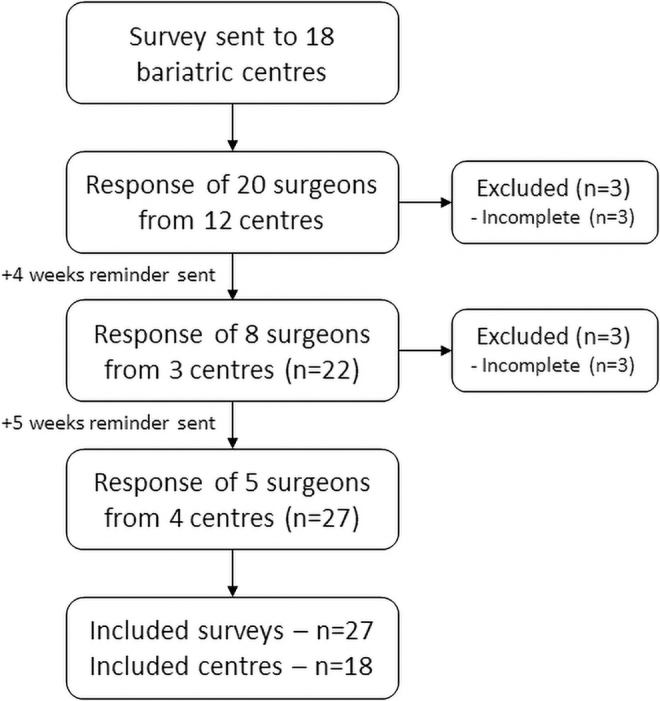
Flowchart of study population selection.

In total, 33 surveys were returned. Of these, six surveys were excluded as they were incomplete. From each bariatric center, at least one survey was completed by a bariatric surgeon (Fig. 1). A median of 4 (range; 2–6) bariatric surgeons were employed per center and 15 (83%) centers had a bariatric surgeon 24/7 available on call. The following three paragraphs present analyzes at surgeon level, whereas the last two paragraphs include analyses at center level:

### Advised type of bariatric procedure

**FIG. 2.**
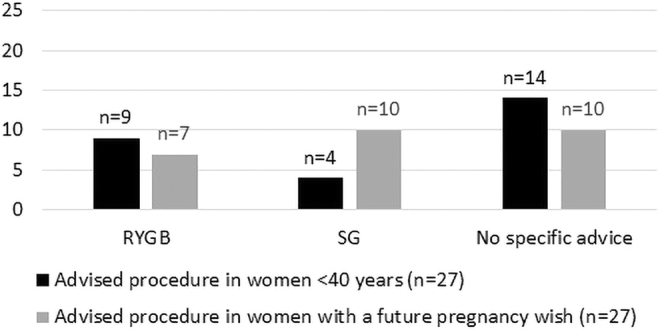
Advised bariatric procedure for fertile women. RYGB, Roux-en-Y gastric bypass; SG, sleeve gastrectomy.

The bariatric procedures that were advised by the respondents are presented in Figure 2. In fertile women (women <40 years), the majority of the surgeons gave no specific advice. Several surgeons, who did not give a specific advice, commented that they based their advice on several factors, including body mass index, comorbidities, and patients' preferences. No surgeon advised a primary banded RYGB, a mini gastric bypass, or a single anastomosis duodenal-ileal bypass with sleeve gastrectomy (SG).

In case of women with a specific future pregnancy wish, the SG became more popular compared to the RYGB, since six surgeons changed their initial advice to a SG. No surgeon changed his/her advice from SG to RYGB.

### Preoperative education

All surgeons provided preoperative education regarding possible bariatric complications related to mother and to unborn child in a future pregnancy after having BS. The recommended time to postpone a pregnancy after BS was 12–18 months (*n* = 17, 63.0%) or 18–24 months (*n* = 10, 37.0%).

### Pregnancy and postpartum counseling

**FIG. 3.**
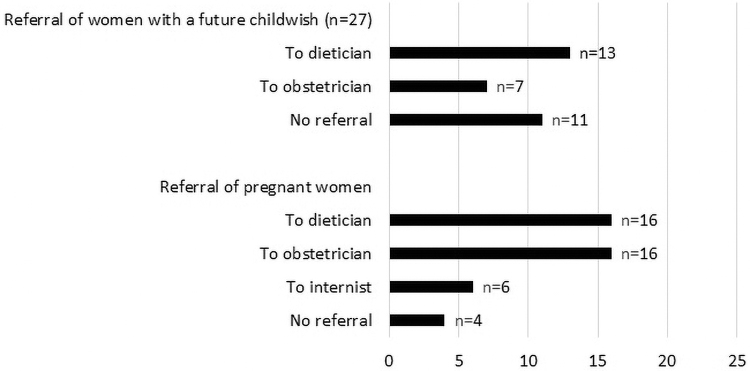
Referral of women with a future child wish and pregnant women after bariatric surgery.

The referral pattern for additional education, counseling, and monitoring of pregnant women or women with a future pregnancy wish after BS is presented in Figure 3. In addition, 15 respondents (55.6%) would invite pregnant patients for an additional consult at their outpatient clinic for information regarding the maternal and fetal risks related to their previous BS. Weight gain during pregnancy was considered acceptable, but only within the normal limits of weight gain during a pregnancy (*n* = 24, 88.9%). Breastfeeding was advised by a vast majority (*n* = 25, 92.6%).

### Diagnosing a pregnant patient with acute abdominal pain

**FIG. 4.**
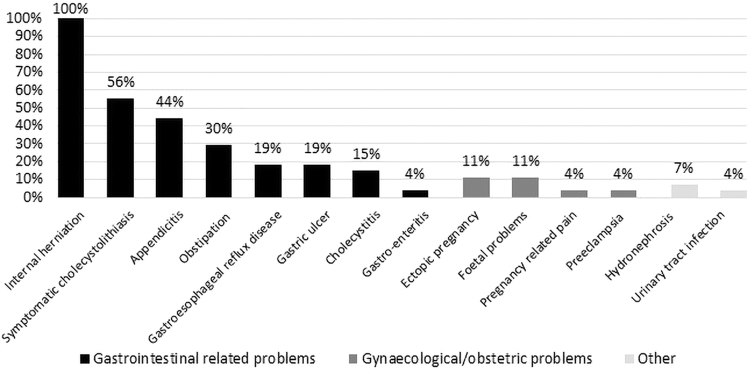
Differential diagnoses in pregnant women with acute abdominal pain after bariatric surgery.

If a pregnant woman presents with acute abdominal complaints after BS, all respondents considered gastrointestinal-related problems as a cause of the abdominal pain and 21 respondents (77.8%) also considered gynecological-related problems. See Figure 4 for the differential diagnoses. No respondent considered intussusception specifically as a differential diagnosis.

The first choice of imaging technique for a pregnant woman with acute abdominal pain was the abdominal ultrasound followed by the magnetic resonance imaging scan (*n* = 21, 77.8%). One respondent preferred the computed tomography scan (CT scan) and another respondent would rather perform an abdominal ultrasound followed by a CT scan.

### Referral/treatment of a pregnant patient with acute abdominal pain

Ten bariatric centers would not refer pregnant women in case of acute abdominal pain. Of these centers, only one is a bariatric center with an OHC and NICU. Furthermore, in one non-NICU bariatric center, only patients with a gestational age (GA) below 24 weeks would be referred to a center with an OHC and NICU (not necessarily a bariatric center). The remaining seven bariatric centers would refer pregnant postbariatric women with acute abdominal pain to a center with an OHC, NICU, and bariatric expertise. Of note, in one bariatric center, different referral choices were noted between surgeons.

Nine of the 18 bariatric centers had a surgical and/or gynecological standard operating procedure (SOP), concerning the diagnosis and therapy of pregnant women who present with acute abdominal pain (two centers were excluded due to discrepant answers between the surgeons). The respondents of the seven centers that lack an SOP indicated that they would prefer to have one.

### Morbidity and mortality

Over the last 5 years, seven (38.9%) bariatric centers have seen at least one or more pregnant women, who previously had BS and presented with acute abdominal pain, with severe morbidity. No case of maternal mortality was reported. Unfortunately, one case of perinatal death in the past 5 years was reported.

## Discussion

Current recommendations regarding the preconception care of women after BS with a child wish are not adhered to. It is recommended to provide preconception care by a bariatric surgeon, dietician, and obstetrician. This is to monitor and prescribe micronutrient supplementation to prevent fetal complications, to inform and educate about possible complications of pregnancy following BS, and to achieve adequate daily protein intake.^17–20^ In this study, which focuses on bariatric surgical points of attention and not gynecologic, 11 surgeons (40.7%) do not refer women with a future child wish to either an obstetrician or a dietician. More attention should be given to this specific preconception care, to prevent pregnancy-related complications.

Several recommendations regarding the care and education of *pregnant women* after BS have been given. Ciangura *et al.*^15^ recommended that the antenatal care should be coordinated by an obstetrician and should include assessment of blood parameters at first presentation and after that, once per trimester. Furthermore, it was recommended to refer pregnant women to a dietician to ensure sufficient energy and micronutrient and protein intake.^15,18^ Further weight loss during pregnancy should be avoided as well as excessive gestational weight gain.^16,21^ Next to this, after labor, breastfeeding is advised with nutritional monitoring and supplementation.^15,16,19,20^ In this study, only nine respondents (33.3%) refer pregnant women after BS to both the obstetrician and dietician. Although many guidelines are available, there is a wide variation in the current practice and the guidelines are often not adhered to. To achieve standardization, there is a need for better information provision for both professionals and patients.

There is no consensus regarding the type of bariatric procedure that should be performed in women with a child wish. RYGB is known for its long-term sustainable weight loss and reduced risks of obesity-related comorbidity.^22–24^ However, it is also associated with many adverse pregnancy-related outcomes.^25,26^ SG is an alternative technique causing less perioperative and long-term complications as well as less adverse pregnancy-related outcomes. However, long-term weight results appear inferior to RYGB and gastroesophageal reflux disease is a major problem.^5,22,25,26^ The most performed technique in the Netherlands is the RYGB.^27^ According to this survey, the RYGB is preferred over SG in fertile women. However, in case of a future child wish, the SG becomes more popular as the primary advised procedure because several surgeons changed their advice from an RYGB or no specific advice to an SG. Ciangura *et al.* concluded that there is yet no evidence available to guide the choice of the most appropriate surgical procedure for fertile women.^15^ Therefore, fertile patients should be educated preoperatively not only regarding the general but also the pregnancy-related benefits and downsides (among others, vitamin deficiencies and acute intestinal complications) of different bariatric procedures. Only then can they make a well-informed decision, taking possible consequences for future pregnancy into account.

The incidence of acute abdominal bariatric complications with maternal or fetal morbidity and mortality is low, but can have disastrous consequences. Literature describes an incidence of internal herniation during pregnancy of as high as 10%, whereas the incidence of intussusception is lower.^10,13^ In 7/18 of the Dutch bariatric centers, maternal morbidity was seen. One case of perinatal death was reported. So only a minority of the Dutch bariatric centers has experience with the severe consequences due to abdominal bariatric complications during pregnancy. Increasing the awareness and sharing the knowledge with surgeons and perinatologists regarding acute abdominal bariatric complications during pregnancy are of importance to provide the best care for these patients.

Referral of pregnant women for acute abdominal bariatric complications to a center with an NICU is not standard of care. Nine bariatric centers, with no OHC and NICU, would not refer postbariatric pregnant patients with acute bariatric complications to an NICU center. This means that some bariatric surgeons would perform surgery in pregnant patients with a GA below 32 weeks, although no specialized care for preterm neonates is available. The reluctance of referral might indicate that bariatric surgeons feel comfortable to take care of this specific group of patients. However, literature has shown multiple cases of significant maternal and/or fetal morbidity and mortality, even when treated in an NICU center.^10,11^ In addition, preterm born infants (<32 weeks gestation) born at an NICU center perform significantly better compared to infants born at a non-NICU-center.^28,29^ Based on these findings, we strongly recommend to refer pregnant patients, 24–32 weeks gestation, who possibly require surgical intervention to a center that has an NICU and an OHC to provide the best perinatal care. We also believe that this should be recommended by the official societies and in consensus statements, especially in countries like the Netherlands, where the distance between the bariatric center with an NICU/OHC and any other bariatric center is (rather) small, exchange of patients is easy.

The main strength of this study is that it is a complete nationwide survey, including all bariatric centers. A limitation is that multiple bariatric surgeons of each bariatric center were not included. Furthermore, many discrepant answers were given between bariatric surgeons, even by those working within the same center. Misinterpretation of a question due to unclear definition is possible, but the discrepancies clearly show that there are different preferences and practices regarding the care of fertile women after BS. In addition, as is inherent in surveys, a response bias is present, although limited due to the development of an anonymous survey.

## Conclusion

This is the first study to investigate the current practice and preferences toward the care that is provided to women with an active child wish before and after BS and to pregnant women after BS. This study has shown that, despite the availability of international guidelines and consensus recommendations regarding the care for these women, there are many differences in the preferences and the current practice among bariatric centers and bariatric surgeons. These discordant practices are an indication for suboptimal care. A multidisciplinary international consensus statement for the treatment of this specific group of patients should be provided, to achieve better information provision for both professionals and patients and thereby provide the best possible care.

## Ethics

The local ethics committee dismissed the study protocol for formal evaluation as it was regarded as a descriptive questionnaire study for which a formal review was not required.

## Consent Statement

All respondents were free to start and complete the survey. No personal information was collected, except for their center of employment. Respondents were informed about the aims of information collection. Completion of the survey was regarded as consent for using the anonymized data in this study.

## Appendix

**Appendix A1.** Pregnancy and Bariatric Surgery: Current Recommendations and Practices in Our Clinic

Preoperatively
Counseling and education regarding the general and pregnancy-related benefits and downsides of different bariatric procedures
Recommendation to avoid pregnancy for twelve months after surgery^A1^
Referral to dietician for adequate monitoring of micronutrient and adequate daily protein intake^A2–A5^
Antenatal care
Referral to dietician to ensure sufficient energy, and micronutrient and protein intake^A3,A6^
Additional multivitamin supplementation (among others; calcium/vitamin D3 500 mg (400 IE)/day and folic acid 400 μg/day) according to Leclercq et al.^A7^
Evaluation and checkups by an obstetrician during the entire pregnancy
Laboratory assessment at first presentation and thereafter once per trimester^,A2,A4–A6,A8^
Additional ultrasound third trimester^A6^
Recommendation to avoid additional weight loss and excessive weight gain^A2,A8^
Referral to facility with neonatal care in case of suspicion of acute bariatric abdominal complication between 24 and 32 weeks of gestation
Vaginal delivery is encouraged^A3^
After childbirth
Lactation is encouraged^A2,A6^
Follow-up with dietician for nutritional supplementation monitoring^A4–A6,A8^

### Appendix A2. Survey Bariatric Surgery and Pregnancy

#### Demographics

1. In which clinic/hospital are you employed?
○ Almere Flevoziekenhuis
○ Amsterdam Obesitas Centrum
○ Arnhem Vitalys Kliniek tegen overgewicht
○ Bergen op Zoom Bariatrisch Centrum Zuid West
○ Beverwijk Nederlandse Obesitas Kliniek
○ Den Haag Nederlandse Obesitas Kliniek West
○ Dordrecht Albert Schweitzer Ziekenhuis
○ Eindhoven Catharina Obesitascentrum
○ Heerlen Nederlandse Obesitas Kliniek Zuid
○ Hengelo ZGT Obesitascenrum
○ Hoofddorp Spaarne Gasthuis
○ Leeuwarden Centrum Obesitas Noord Nederland
○ Nieuwegein Nederlandse Obesitas Kliniek
○ Rotterdam Obesitas Centrum Franciscus Gasthuis and Vlietland
○ Terneuzen Bariatrisch Centrum Zorgsaam
○ Tilburg Obesitas Centrum Midden Brabant
○ Veldhoven Obesitas Centrum Máxima
2. What is your current working position/job title?
○ Surgical resident
○ PhD student
○ Surgeon
○ Physician assistant/nurse practitioner
○ Other, namely….
3. How many bariatric surgeons are currently employed in your clinic/hospital?
(Permanent employees include bariatric surgeons and fellows working bariatric shifts)
○ 2
○ 3
○ 4
○ 5
○ 6
○ 7
○ 8
○ 9
○ 10
4. Are the bariatric surgical consultants in your clinic/hospital 24/7 available on call?
○ Yes
○ No
○ I do not know
5. Does your clinic/hospital have an obstetric high care (OHC)?
○ Yes
○ No
○ I do not know
6. Does your clinic/hospital have a neonatal intensive care unit (NICU)?
○ Yes
○ No
○ I do not know

#### Preconceptual

7. Is preoperative counseling provided concerning the potential bariatric complications in a future pregnancy?
○ Yes
○ No
8. What type of bariatric surgery do you generally advise in women who are <40 years old?
○ Sleeve gastrectomy
○ Gastric bypass
○ No specific advice, comment….
○ Other, namely….
9. Does the advice change if the patient has a future pregnancy wish?
○ Yes, to a sleeve gastrectomy
○ Yes, to a gastric bypass
○ No change
○ No specific advice, comment….
○ Other, namely….
10. Do you standardly refer patients after bariatric surgery, who have a future child wish, to a gynecological gynecologist?
○ Yes
○ No
11. Do you standardly refer patients after bariatric surgery with a child wish to a dietitian?
○ Yes
○ No
12. How long after bariatric surgery would you recommend a patient to wait with a pregnancy?
○ The patient does not need to wait
○  < 1 year
○ 1–1.5 years
○ 1.5–2 years
○  > 2.5 years

#### Pregnancy

The following questions concern the scenario where a pregnant woman is seen after having undergone bariatric surgery.

13. If a pregnant patient presents in your bariatric clinic/hospital, does she receive an extra checkup at the bariatric surgery department to provide counseling on maternal and fetal risks?
○ Yes
○ No
○ Other, namely….
14. Do you normally refer a pregnant patient to a gynecologist?
○ Yes
○ No
15. Do you normally refer a pregnant patient to an internal medicine specialist?
○ Yes
○ No
16. Do you normally refer a pregnant patient to a dietician?
○ Yes
○ No
17. What is your advice on body weight for a pregnant patient during her pregnancy?
○ No advice is given
○ Maintain the same weight
○ Weight gain is acceptable, but only within the normal limits of weight gain during a pregnancy
○ Weight gain is no problem
18. Does your clinic/hospital have a protocol for the diagnosis and therapy of a pregnant patient who presents with acute abdominal pain?
○ Yes, the surgery department has a protocol
○ Yes, the gynecology department has a protocol
○ Yes, both the surgery and the gynecology department have a protocol
○ No, but there is need for one
○ No, and there is no need for one
19. If a pregnant patient presents with acute abdominal pain, which diagnosis would be in the top of the list of your differential diagnosis?
(Give at least 3 answers)
○ Diagnosis 1…….
○ Diagnosis 2.…….
○ Diagnosis 3…….
○ Diagnosis 4…….
○ Diagnosis 5…….
20. When do you refer a pregnant patient with acute abdominal complaints to another clinic/hospital?
○ When the gestational age is <24 weeks
○ When the gestational age is <32 weeks
○ When the gestational age is <37 weeks
○ I refer everyone, regardless of the gestational age
○ I do not refer these patients
21. To what kind of center do you refer these patients to?
○ To a bigger bariatric center
○ To a center with OHC and NICU
○ To a center with OHC and NICU and what has bariatric expertise
○ Other, namely….
22. If a pregnant patient presents with acute abdominal complaints, what would be your first choice of imaging techniques?
○ An abdominal ultrasound only
○ An abdominal ultrasound followed by computed tomography scan (CT scan)
○ An abdominal ultrasound followed by CT scan and magnetic resonance imaging scan (MRI scan)
○ An abdominal ultrasound followed by MRI scan
○ A CT scan only
○ CT scan followed by abdominal ultrasound
○ MRI scan only
○ Other, namely….

#### Morbidity and mortality

The following questions concern the scenario where a pregnant woman is seen after having undergone bariatric surgery.

23. Has a *pregnant patient with severe maternal morbidity* presented herself with acute abdominal complaints at least once in the past five years in your bariatric center? For example, presentation with an ischemic small bowel that needed resection?
○ Yes
○ No
24. Has *maternal* mortality occurred in a pregnant patient with acute abdominal complaints as consequence of a *bariatric complication* in the last 5 years in your bariatric center (at least once)?
○ Yes
○ No
25. Has *fetal mortality* occurred in a pregnant patient with acute abdominal complaints as consequence of a *bariatric complication* in the last 5 years in your bariatric center (at least once)?
○ Yes
○ No

#### Postpartum

26. What is your advice on breastfeeding to postbariatric patients?
○ Breastfeeding is preferred
○ Preferably no breastfeeding
○ Breastfeeding is strongly discouraged
27. Does you clinic/hospital provide standard postpartum counseling at the bariatric surgery department?
○ I do not know
○ No, only regular follow-up
○ Yes, what subjects are discussed;…….
